# Differential Effects of *Opuntia ficus-indica* and *Opuntia stricta* var. *dillenii* Extracts on Liver Steatosis in a Murine Dietary Model

**DOI:** 10.3390/nu18030420

**Published:** 2026-01-27

**Authors:** Irene Besné-Eseverri, Denis Benito, Miren Fisico-Echezarraga, Miguel Arán-González, M. Pilar Cano, María P. Portillo, Jenifer Trepiana

**Affiliations:** 1 Nutrition and Obesity Group, Department of Nutrition and Food Sciences, Faculty of Pharmacy, University of the Basque Country (UPV/EHU), Lucio Lascaray Research Centre, 01006 Vitoria-Gasteiz, Spain; irene.besne@ehu.eus (I.B.-E.); mirenfisico@gmail.com (M.F.-E.); mariapuy.portillo@ehu.eus (M.P.P.); 2 CIBER Physiopathology of Obesity and Nutrition (CIBERobn), Institute of Health Carlos III, 28029 Madrid, Spain; 3 CBET+ Research Group, Department of Zoology and Animal Cell Biology, Faculty of Science and Technology, Research Centre for Experimental Marine Biology and Biotechnology PiE-UPV/EHU, University of the Basque Country UPV/EHU, Areatza z/g, 48620 Plentzia, Spain; denis.benito@ehu.eus; 4 Unidad de Gestión Clínica de Anatomía Patológica de Guipúzcoa, Hospital Universitario Donostia, 20014 San Sebastián, Spain; miguel.arangonzalez@osakidetza.eus; 5 Laboratory of Phytochemistry and Plant Food Functionality, Biotechnology and Food Microbiology Department, Institute of Food Science Research (CIAL) (CSIC-UAM), Nicolás Cabrera 9, 28049 Madrid, Spain; mpilar.cano@csic.es; 6 BIOARABA Health Research Institute, 01006 Vitoria-Gasteiz, Spain

**Keywords:** MASLD, steatosis, *Opuntia ficus-indica* var. *colorada*, *Opuntia stricta* var. *dillenii*, lipogenesis *de novo*

## Abstract

**Background/Objectives**: Metabolic-dysfunction-associated fatty liver disease (MASLD) is characterised by an excessive hepatic lipid accumulation. The present research aims to study the impact of an *Opuntia stricta* var. *dillenii* peel extract and an *Opuntia ficus-indica* var. *colorada* pulp extract, known for their high content of betalains and phenolic compounds, on the prevention of hepatic lipid accumulation in the liver of rats with steatosis. **Methods**: For this, MASLD was induced in 60 male Wistar rats by a high-fat high-fructose diet. They were supplemented with *Opuntia stricta* var. *dillenii* peel extract or *Opuntia ficus-indica* var. *colorada* pulp extract at 25 or 100 mg/kg body weight/d. **Results**: The high-fat high-fructose diet caused an increase in final body and liver weight, hepatic triglyceride (TG) content, and altered liver histology. The increase in hepatic TG was due to the rise in fatty acid uptake and the increased assembly of TG, although increased *de novo* lipogenesis cannot be ruled out. The treatment with a low dose of *Opuntia ficus-indica* var. *colorada* pulp extract (L-OFI group) significantly prevented hepatic TG accumulation, and the high dose (H-OFI group) showed a tendency towards lower values compared to the rats fed the high-fat high-fructose diet. The main mechanism of action appears to be a down-regulation of fatty acid uptake. By contrast, *Opuntia stricta* var. *dillenii* peel extract did not prevent the high-fat high-fructose diet-induced steatosis. **Conclusions**: Overall, *Opuntia ficus-indica* var. *colorada* pulp extract may represent a potential strategy for MASLD prevention, although its beneficial effects require confirmation in human studies.

## 1. Introduction

Metabolic-dysfunction-associated steatotic liver disease (MASLD) refers to a condition characterised by hepatic steatosis (more than 5% of hepatocytes containing fat) linked to type 2 diabetes mellitus and overweight or obesity, in the absence of significant alcohol consumption or other chronic liver diseases [1,2]. Recent reports indicate that the prevalence of MASLD exceeds 30% of the overall population [3], and that the disease can progress from simple fatty liver to more severe stages, including metabolic-dysfunction-associated steatohepatitis (MASH) and cirrhosis, potentially complicated by hepatocellular carcinoma (HCC) [3,4].

The intake of high-fat diets, particularly those rich in saturated fats, was initially considered the primary driver of obesity and MASLD, characteristic of Western dietary patterns [5,6]. More recently, the consumption of fructose has been demonstrated to promote lipogenesis, potentially to a greater extent than high-fat diets, due to specific features of fructose metabolism [5,7]. As no medication has been specifically approved for the treatment of MASLD, dietary modifications and physical exercise are generally considered the cornerstone of management [8]. However, accomplishing these lifestyle modifications can be challenging due to poor adherence, highlighting the need for new strategies to manage MASLD. Particular attention has been paid to plants and foods rich in bioactive compounds, which have been proven to be beneficial in the modulation of key enzymes involved in MASLD [9].

*Opuntia*, a member of the Cactaceae family that thrives in arid and desert habitats, is a rich source of bioactive substances, including betalains (betacyanins and betaxanthins), phenolic compounds (flavonoids and phenolic acids), carotenoids, vitamins, and fibre. The proportion of these compounds varies according to the specific plant tissue examined [10,11]. Extracts and derived products from several *Opuntia* species have demonstrated promising hepatoprotective potential; however, most investigations to date have primarily focused on *Opuntia ficus-indica* [12]. *Opuntia stricta* var. *dillenii*, a comparatively underexplored species, is distributed across regions such as Spain (Canary Islands, Murcia, and Almeria), Italy, India, and parts of Africa. This species is distinguished by its high betalain content, with betanin being the dominant pigment responsible for the characteristic coloration of the fruit [13,14]. In a previous study conducted by our research group, the effects of extracts derived from the whole fruit, pulp, peel, and bagasse of *Opuntia ficus-indica* and *Opuntia stricta* var. *dillenii* on triglyceride accumulation were evaluated using an *in vitro* model of liver steatosis. The findings indicated that the most effective extracts were the pulp of *Opuntia ficus-indica* var. *colorada* and the peel of *Opuntia stricta* var. *dillenii* [15,16]. In this context, the present study aims to examine the effects of both extracts on the prevention of diet-induced MASLD in rats, as well as to elucidate the pathways related to lipid metabolism affected by these treatments.

## 2. Materials and Methods

### 2.1. Opuntia stricta var. dillenii and Opuntia ficus-indica var. colorada Extracts

Prickly pear fruits of *Opuntia stricta* var. *dillenii* were harvested in Tenerife (28°32′03″ N, 16°23′50″ W above sea level, Canary Islands, Spain), while fruits of *Opuntia ficus-indica* var. *colorada* (Spanish orange variety) were sourced from Fasnia (28°14′44″ N, 16°26′10″ W; 446 m above sea level, Tenerife, Canary Islands, Spain) [17].

Aqueous prickly pear extracts were prepared from freeze-dried plant material through sequential extractions using methanol–water (1:1, *v*:*v*), followed by methanol, yielding extracts high in betalains and phenolic compounds [18]. The resulting extracts were lyophilised, and stock solutions (200 mg/mL in water) were formulated, aliquoted, and stored at −20 °C until administration to the animals.

As shown in Table 1, although both *Opuntia* varieties are rich sources of betalains and phenolic compounds, significant compositional differences exist between them. The peel of *Opuntia stricta* var. *dillenii* contains elevated contents of betanin, isobetanin, phyllocactin, and neobetanin (betalains), as well as piscidic acid (phenolic acid). Conversely, the pulp of *Opuntia ficus-indica* var. *colorada* is abundant in indicaxanthin (betalain) and contains piscidic acid at much lower levels than the *Opuntia stricta* var. *dillenii* extract. Other betaxanthins are also present in this extract, albeit at smaller concentrations.

### 2.2. Animals, Diets, and Experimental Design

Sixty male Wistar rats (4 weeks old; 125–145 g) were obtained from Envigo (Barcelona, Spain) and maintained in accordance with institutional protocols for the care and use of laboratory animals (M20_2022_283). The rats were housed in pairs in polycarbonate cages in a temperature-controlled room (22 ± 2 °C) under a 12 h light–dark cycle. Following a week-long acclimatisation period, the animals were assigned to six experimental groups: the control group (C group; *n* = 10) was fed a standard commercial diet (D10012G; Research Diets, New Brunswick, NJ, USA), whereas all remaining groups received a high-fat high-fructose diet (D21052401; Research Diets, New Brunswick, NJ, USA; Table 2) over eight weeks. Subsets within the high-fat high-fructose diet groups were simultaneously administered, while maintained on the HFHF diet, a daily oral solution containing 2.5% sucrose and either *Opuntia stricta* var. *dillenii* peel extract at a low (25 mg/kg body weight, L-OD group; *n* = 10) or high (100 mg/kg body weight, H-OD group; *n* = 10) dose, or *Opuntia ficus-indica* var. *colorada* pulp extract at a low (25 mg/kg body weight, L-OFI group; *n* = 10) or high (100 mg/kg body weight, H-OFI group; *n* = 10) dose.

Food was withheld for 12 h prior to the conclusion of the experimental period. The final administration of the treatments was carried out 3 h before euthanasia, which was performed under chloral hydrate anaesthesia by cardiac exsanguination. Livers were excised, weighed, and immediately frozen in liquid nitrogen. All samples were stored at −80 °C until further analysis.

### 2.3. Liver Triglyceride Content, Hepatic Cholesterol Levels, and Hepatic Index

Total lipids from liver tissue were extracted using the method described by Folch et al. [19]. The resulting lipid extract was reconstituted in isopropanol, and triglyceride levels were quantified spectrophotometrically using a commercial assay kit (Spinreact, Barcelona, Spain). Concerning hepatic cholesterol, it was measured spectrophotometrically employing a commercial kit (BioSystems, Barcelona, Spain). The hepatic index was calculated as the ratio of liver weight/body weight × 100.

### 2.4. Liver Histology

Immediately following sacrifice, a liver section from the same lobe of each animal was fixed in 10% buffered formalin and embedded in paraffin. Sections were then stained with haematoxylin and eosin following standard procedures. Hepatic fat accumulation was graded according to the classification of Brunt et al. [20]: Grade 0, no fat; Grade 1, fat vacuoles in <33% of hepatocytes; Grade 2, fat vacuoles in 33–66% of hepatocytes; Grade 3, fat vacuoles in >66% of hepatocytes. All samples were independently evaluated by two experienced pathologists blinded to the experimental groups, who reached a consensus on the grading.

### 2.5. Enzymatic Activities of Proteins Related to Lipogenesis and Fatty Acid Oxidation

Liver samples (100–150 mg) were homogenised in 1 mL of buffer (250 mM sucrose, 1 mM EDTA, 10 mM Tris–HCl, pH 7.4) and centrifuged at 700× *g* for ten minutes at 4 °C. The resulting supernatant was collected and further centrifuged again at 12,000× *g* for 15 min at 4 °C to obtain the cytosolic fraction. Pellets containing the mitochondrial fraction were resuspended in a solution of 70 mM saccharose, 220 mM mannitol, 2 mM HEPES, and 1 mM EDTA at pH 7.4. Protein levels in both fractions were determined using the Bradford method [21] with bovine serum albumin (BSA) as the standard.

*De novo* lipogenesis was assessed by measuring fatty acid synthase (FAS) activity in the cytosolic fraction, following the methods of Lynen [22] and Nepokroeff et al. [23]. FAS activity was measured by monitoring changes in absorbance associated with the consumption of nicotinamide adenine dinucleotide phosphate (NADPH) during the enzymatic reaction and expressed as consumed nmol/minXmg protein. Activities of glucose 6-phosphatase and malic enzyme, which provide substrates for the lipogenic pathway, were also assessed. Glucose 6-phosphatase activity was measured according to Kuby and Noltmann [24], based on the formation of NADPH and expressed as nmol NADPH/minXmg protein. Similarly, malic enzyme activity was determined following Hsu and Lardy [25], with absorbance changes reflecting NADPH production. The endpoints were expressed as generated NADPH/minXmg protein.

The function of enzymes related to fatty acid oxidation was evaluated using the mitochondrial/peroxisomal fraction. Carnitine palmitoyl transferase-1a (CPT1A), which mediates the transport of long-chain fatty acyl-CoA into mitochondria for β-oxidation, was evaluated spectrophotometrically using the technique outlined by Bieber et al. [26]. This approach relies on measuring the released CoA-SH, with results expressed as nmol/minXmg protein. Citrate synthase, a marker of mitochondrial content [27], was assessed spectrophotometrically through quantification of free CoA formation, according to Srere [28]. Citrate synthase activity was determined after incubating samples at 30.1 °C for two minutes in a solution containing acetyl-CoA, 1.01 mM DTNB, 10 mM oxaloacetate, Triton X-100 (10%), and distilled water. Absorbance was recorded at 412 nm initially and again following a 5 min incubation. Citrate synthase activity was determined by calculating the difference between the two absorbance readings and expressed as nmol CoA/minXmg protein.

### 2.6. Immunoblot Analysis of Proteins Involved in Hepatic Lipid Metabolism

Liver homogenates were prepared using 100 mg of tissue in 1.5 mL of PBS (0.15 M NaCl, 3 mM KCl, 3 mM NaH_2_PO_4_, 7.5 mM Na_2_HPO_4_; pH 7.4), supplemented with protease inhibitors (1 mM phenylmethylsulphonyl fluoride and 0.1 mM iodoacetamide). Homogenates were centrifuged at 800× *g* for five minutes at 4 °C, and the resulting supernatants were used for protein expression analysis by Western blot. Total protein concentrations were determined spectrophotometrically at 595 nm using the Bradford assay [21], with BSA as the standard.

Total protein homogenates were analysed for the expression of fatty acid transport protein 2 (FATP2), fatty acid transport protein 5 (FATP5), cluster of differentiation 36 (CD36), aquaporin 9 (AQP9), phosphorylated acetyl-CoA carboxylase (pACC), total acetyl-CoA carboxylase (ACC), fatty acid synthase (FAS), phosphorylated salt inducible kinase 1 (pSIK1), total salt inducible kinase 1 (SIK1), diacylglycerol O-acyltransferase (DGAT2), phosphorylated AMP-activated protein kinase (pAMPK), total AMP-activated protein kinase (AMPK), sirtuin-1 (SIRT1), mitochondrial transcription factor A (TFAM), carnitine palmitoyl transferase-1a (CPT1A), mitochondrial uncoupling protein 2 (UCP2), abhydrolase domain containing 5, lysophosphatidic acid acyltransferase (ABHD5), adipose triglyceride lipase (ATGL), microsomal triglyceride transfer protein (MTP), phosphorylated unc-51-like autophagy activating kinase 1 (pULK1), total unc-51-like autophagy activating kinase 1 (ULK1), microtubule-associated protein 1A/1B-light chain 3 (LC3), sequestosome-1 (p62), and glyceraldehyde 3-phosphte dehydrogenase (GAPDH). Nuclear protein extracts were prepared from 150–200 mg of liver tissue for the analysis of sterol regulatory element-binding transcription factor 1 (SREBP-1c), carbohydrate-responsive element-binding protein (ChREBP), acetylated lysine, total peroxisome proliferator-activated receptor-gamma coactivator 1α (PGC-1α), nuclear respiratory factor 1 (NRF1), and histone H3, adhering to established methods [29].

Immunoblot studies were performed using 60 µg of total liver protein, denatured for five minutes at 95 °C in Laemmli buffer [30]. Proteins were separated via electrophoresis on 4–15% SDS-polyacrylamide gels and subsequently transferred to polyvinylidene difluoride (PVDF) membranes (Merck, Darmstadt, Germany). Membranes were incubated with 4% BSA for 1.5 h at room temperature, followed by overnight incubation at 4 °C with primary antibodies (1:1000) against FATP2 (Santa Cruz Biotech, Dallas, TX, USA), FATP5 (Lifespan Bioscience, Lynnwood, DC, USA), CD36 (Cell Signaling Danvers, MA, USA), AQP9 (Santa Cruz Biotech, Dallas, TX, USA), ChREBP (Novus Biologicals, Centennial, CO, USA), SREBP-1c (Abcam, Cambridge, UK), pACC (1Cell Signaling Danvers, MA, USA), ACC (Cell Signaling Danvers, MA, USA), FAS (Abcam, Cambridge, UK), pSIK1 (Thermo Fisher Scientific Inc., Rockford, IL, USA), SIK1 (Novus Biologicals, Centennial, CO, USA), DGAT2 (Abcam, Cambridge, UK), pAMPK (Cell Signaling Danvers, MA, USA), AMPK (Cell Signaling Danvers, MA, USA), SIRT1 (Abcam, Cambridge, UK), acetylated lysine (Cell Signaling Danvers, MA, USA), PGC-1α (1Novus Biologicals, Centennial, CO, USA), NRF1 (Abcam, Cambridge, UK), TFAM (Santa Cruz Biotech, Dallas, TX, USA), CPT1A (Abcam, Cambridge, UK), UCP2 (Santa Cruz Biotech, Dallas, TX, USA), ABHD5 (Invitrogen, Waltham, MA, USA), ATGL (Cell Signaling Danvers, MA, USA), MTP (Abcam, Cambridge, UK), pULK1 (Ser 757; Cell Signaling Danvers, MA, USA), ULK1 (Abcam, Cambridge, UK), LC3 (Cell Signaling Danvers, MA, USA), p62 (Abcam, Cambridge, UK), GAPDH (Abcam, Cambridge, UK), and histone H3 (Abcam, Cambridge, UK). Afterwards, membranes were incubated for 2 h at room temperature with the corresponding secondary antibodies (1:5000): polyclonal anti-mouse for AQP9, CPT1A, UCP2, p62, and GAPDH; anti-rabbit for FATP5, CD36, ChREBP, SREBP-1c, pACC, ACC, FAS, pSIK1, SIK1, DGAT2, pAMPK, AMPK, SIRT1, acetylated lysine, PGC-1α, NRF1, ABHD5, ATGL, MTP, pULK1, ULK1, LC3, and histone H3; and anti-goat for FATP2 and TFAM (all from Santa Cruz Biotech, Dallas, TX, USA). Bound antibodies were detected using an ECL system (Thermo Fisher Scientific Inc., Rockford, IL, USA) and quantified with a ChemiDoc MP Imaging System (Bio-Rad, Hercules, CA, USA). Protein expression levels were standardised against GAPDH, histone H3, or the corresponding phosphorylated isoforms.

### 2.7. Statistical Analysis

Data are expressed as mean ± SEM. Statistical analyses were carried out using SPSS 24.0 (SPSS, Chicago, IL, USA). Data normality was assessed by the Shapiro–Wilk test. Comparisons between groups receiving low or high doses of each *Opuntia* extract (*Opuntia stricta* var. *dillenii* or *Opuntia ficus-indica* var. *colorada*) and the control and the HFHF groups were conducted using one-way ANOVA followed by the Newman–Keuls *post hoc* test. Differences were considered statistically significant at *p* < 0.05.

## 3. Results

### 3.1. Final Body Weight and Energy Intake

**Figure 1:**
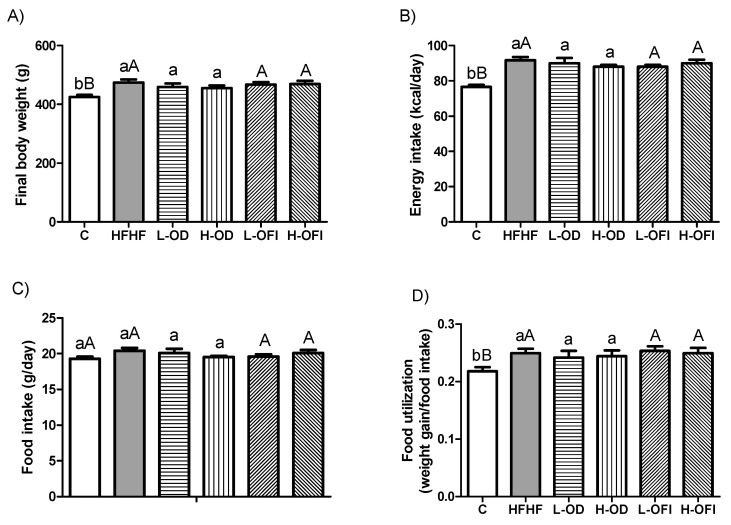
Final body weight (g) (**A**), energy intake (kcal/day) (**B**), food intake (g/day) (**C**), and food utilisation (**D**) of rats fed a control diet (C), a high-fat high-fructose diet alone (HFHF), or supplemented with low or high doses of *Opuntia stricta* var. *dillenii* peel extract (L-OD and H-OD, respectively) or *Opuntia ficus-indica* var. *colorada* pulp extract (L-OFI and H-OFI, respectively) for 8 weeks. Data are presented as mean ± SEM. Group differences were assessed using one-way ANOVA followed by the Newman–Keuls *post hoc* test. Bars not sharing a common letter indicate statistically significant differences (*p* < 0.05). Lowercase letters denote comparisons among C, HFHF, L-OD, and H-OD groups, whereas uppercase letters denote comparisons among C, HFHF, L-OFI, and H-OFI groups.

Final body weight and daily energy intake were measured. Rats in the HFHF group exhibited significantly greater final body weight than control animals receiving the standard diet, whereas *Opuntia* supplementation did not reduce body weight relative to the HFHF group (Figure 1A). Although daily food intake was similar across all groups, rats receiving the high-fat high-fructose diet had higher calorie intake than controls, regardless of *Opuntia* supplementation (Figure 1B,C). In the same line, the food efficiency was increased in rats fed the HFHF diet, with this parameter not being modified by the extracts (Figure 1D).

### 3.2. Liver Weight, Hepatic Index, Liver Triglyceride, Cholesterol Content, and Steatosis Grade

**Figure 2:**
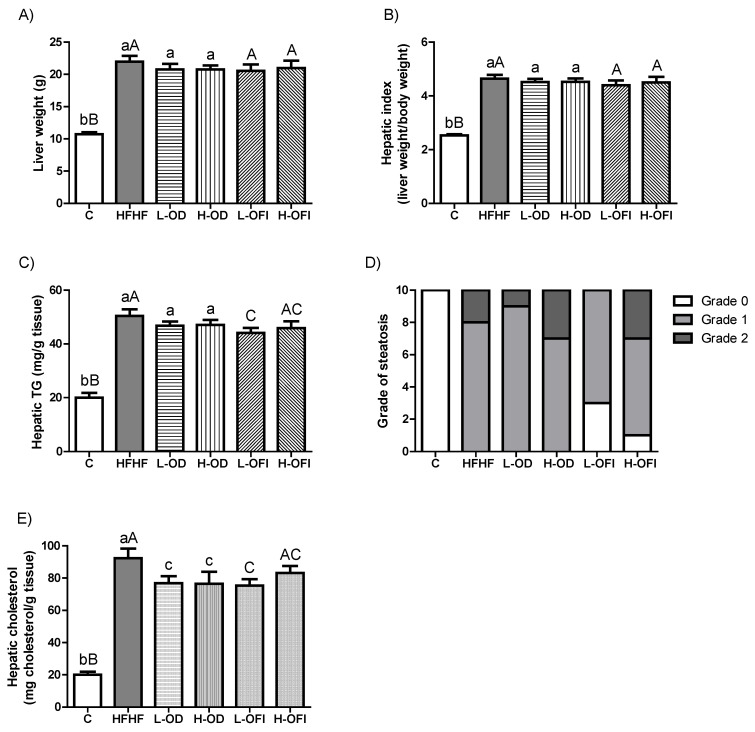
Liver weight (g) (**A**), hepatic index (**B**), hepatic TG (mg/g tissue) (**C**), steatosis grade (**D**), and hepatic cholesterol content (mg/g tissue) (**E**) in rats fed a control diet (C), a high-fat high-fructose diet alone (HFHF), or supplemented with low or high doses of *Opuntia stricta* var. *dillenii* peel extract (L-OD and H-OD, respectively) or *Opuntia ficus-indica* var. *colorada* pulp extract (L-OFI and H-OFI, respectively) for 8 weeks. Data are presented as mean ± SEM. Group differences were assessed using one-way ANOVA followed by the Newman–Keuls *post hoc* test. Bars not sharing a common letter indicate statistically significant differences (*p* < 0.05). Lowercase letters denote comparisons among C, HFHF, L-OD, and H-OD groups, whereas uppercase letters denote comparisons among C, HFHF, L-OFI, and H-OFI groups. TG: triglyceride.

**Figure 3:**
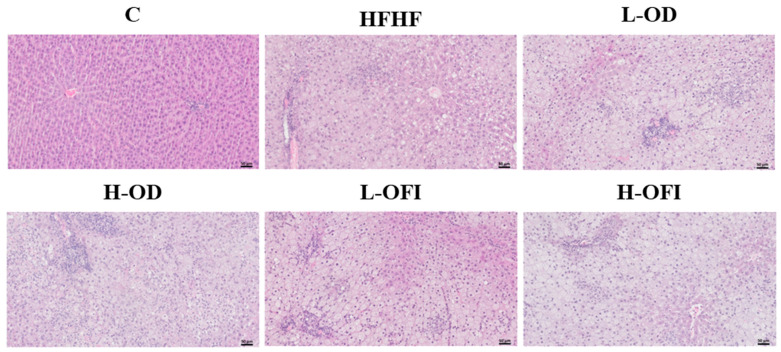
Representative liver sections stained with haematoxylin and eosin from rats fed a standard diet (C), a high-fat high-fructose diet alone (HFHF), or supplemented with low or high doses of *Opuntia stricta* var. *dillenii* peel extract (L-OD and H-OD, respectively) or *Opuntia ficus-indica* var. *colorada* pulp extract (L-OFI and H-OFI, respectively) for 8 weeks. Images are shown at 200× magnification.

To assess steatosis in this in vivo model of MASLD, liver weight, hepatic index, hepatic triglyceride and cholesterol content, and steatosis grade were determined. Rats fed the HFHF diet showed significantly increased liver weight (*p* < 0.001), hepatic index (*p* < 0.001), and hepatic triglycerides (*p* < 0.001) compared to control animals (Figure 2A–C). Histopathological analysis, as expected, showed that control animals had Grade 0 steatosis, whereas 80% of HFHF rats had Grade 1 and 20% Grade 2 steatosis (Figure 2D and Figure 3). Additionally, HFHF feeding significantly enhanced hepatic cholesterol content (Figure 2E).

Concerning *Opuntia stricta* var. *dillenii* peel extract, neither the low nor the high dose modified liver weight, hepatic index, hepatic triglyceride content, or steatosis grade compared to the HFHF group (Figure 2A–D and Figure 3). In contrast, both doses significantly lowered hepatic cholesterol levels (Figure 2E). In the case of the *Opuntia ficus-indica* var. *colorada* pulp extract, the L-OFI treatment significantly reduced hepatic triglyceride content by 12.5% compared to the HFHF group (Figure 2C) and significantly decreased hepatic cholesterol levels (Figure 2E). Histological analysis further indicated an improvement in steatosis grade, since 30% of animals in this group displayed Grade 0, while the reminder had Grade 1 steatosis (Figure 2D and Figure 3). In the H-OFI group, a reduction in hepatic triglyceride levels was also noted, amounting to 8.9% compared to the HFHF group (Figure 2C), together with a modest improvement in steatosis grade (Figure 2D and Figure 3).

### 3.3. Enzymatic Activities and Expression of Proteins Related to Liver Lipid Metabolism

#### 3.3.1. Proteins Involved in Lipid and Glycerol Uptake in the Liver

**Figure 4:**
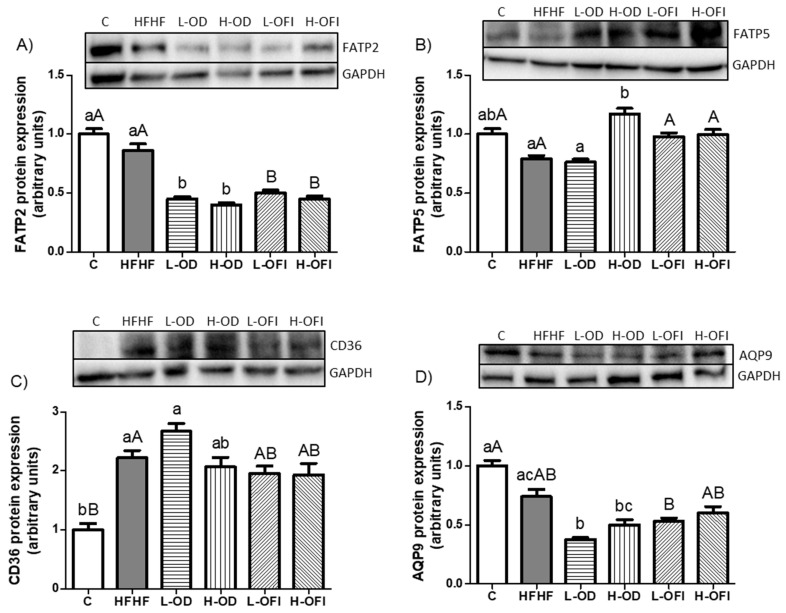
Hepatic protein expression of FATP2 (**A**), FATP5 (**B**), CD36 (**C**), and AQP9 (**D**) in rats fed a control diet (C), a high-fat high-fructose diet alone (HFHF), or supplemented with low or high doses of *Opuntia stricta* var. *dillenii* peel extract (L-OD and H-OD, respectively) or *Opuntia ficus-indica* var. *colorada* pulp extract (L-OFI and H-OFI, respectively) for 8 weeks. Data are presented as mean ± SEM. Differences among groups were analysed using one-way ANOVA followed by the Newman–Keuls *post hoc* test. Bars not sharing a common letter indicate statistically significant differences (*p* < 0.05). Lowercase letters denote comparisons among C, HFHF, L-OD, and H-OD groups, whereas uppercase letters denote comparisons among C, HFHF, L-OFI, and H-OFI groups. AQP9: Aquaporin 9; CD36: cluster of differentiation 36; FATP2: fatty acid transport protein 2; FATP5: fatty acid transport protein 5.

HFHF feeding did not alter FATP2 or FATP5 expression (Figure 4A,B). In contrast, CD36 expression was significantly increased (Figure 4C). Since glycerol availability is required for triglyceride formation, AQP9 expression was also assessed. In this case, no significant differences were noted between the HFHF and the control groups (Figure 4D).

Concerning *Opuntia stricta* var. *dillenii* peel extract, the low dose significantly reduced FATP2 (Figure 4A) and AQP9 expression (Figure 4D) compared to the HFHF treatment. In the H-OD group, FATP2 levels were also significantly decreased (Figure 4A), whereas FATP5 expression was significantly enhanced (Figure 4B) relative to the HFHF-fed animals. Similarly, supplementation with *Opuntia ficus-indica* var. *colorada* pulp extract resulted in a significant down-regulation of FATP2 expression at both low and high doses relative to the HFHF group (Figure 4A).

#### 3.3.2. Activity of Enzymes and Expression of Proteins and Transcription Factors Relevant to *De Novo* Lipogenesis

**Figure 5:**
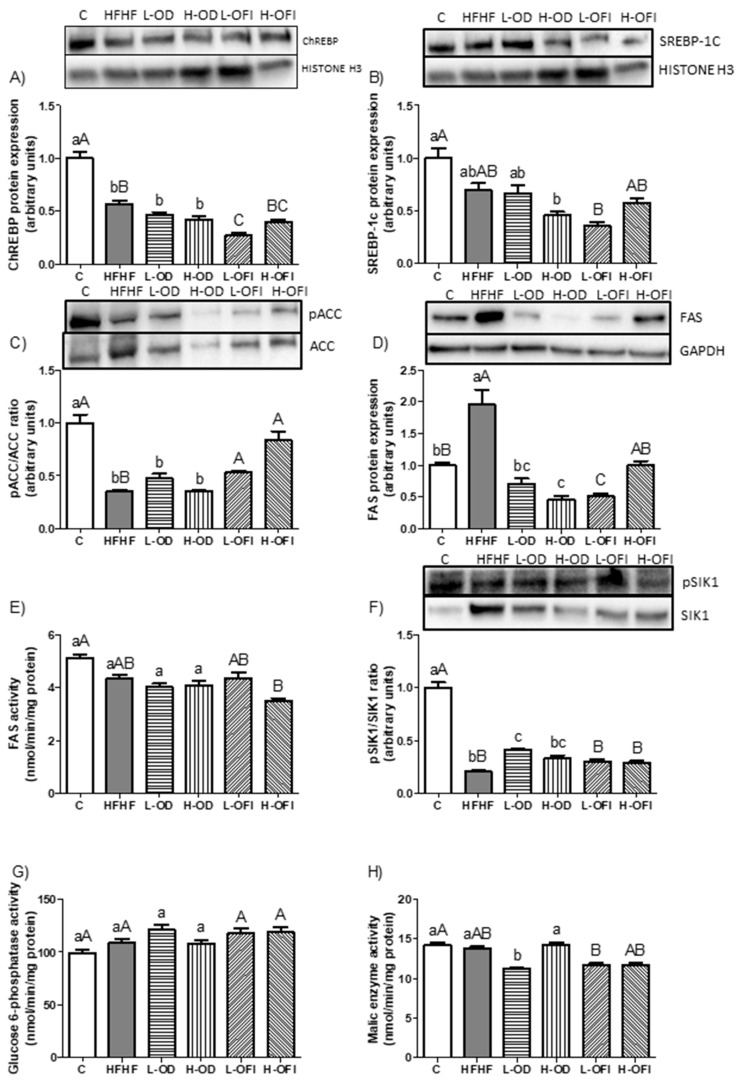
Hepatic protein expression levels of ChREBP (**A**), SREBP-1c (**B**), pACC/ACC ratio (**C**), FAS (**D**), FAS activity (**E**), pSIK1/SIK1 ratio (**F**), glucose 6-phosphatase activity (**G**), and malic enzyme activity (**H**) in rats fed a control diet (C), a high-fat high-fructose diet alone (HFHF), or supplemented with low or high doses of *Opuntia stricta* var. *dillenii* peel extract (L-OD and H-OD, respectively) or *Opuntia ficus-indica* var. *colorada* pulp extract (L-OFI and H-OFI, respectively). Data are presented as mean ± SEM. Differences among groups were analysed using one-way ANOVA followed by the Newman–Keuls *post hoc* test. Bars not sharing a common letter indicate statistically significant differences (*p* < 0.05). Lowercase letters denote comparisons among C, HFHF, L-OD, and H-OD groups, whereas uppercase letters denote comparisons among C, HFHF, L-OFI, and H-OFI groups. ACC: acetyl-CoA carboxylase; ChREBP: carbohydrate-responsive element-binding protein; FAS: fatty acid synthase; SIK1: salt inducible kinase 1; SREBP-1c: sterol regulatory element-binding transcription factor 1c.

To investigate the *de novo* lipogenesis pathway, enzymatic activity and/or protein expression of key enzymes were assessed. ChREBP and SREBP-1c, two transcription factors regulating ACC and FAS, were analysed. ChREBP expression was significantly reduced in the HFHF group compared to control group (Figure 5A), whereas no differences were detected in SREBP-1c levels (Figure 5B). The pACC/ACC ratio was significantly reduced in the HFHF group compared to control animals (Figure 5C), while FAS expression was significantly increased (Figure 5D). In contrast, FAS enzymatic activity did not change (Figure 5E). The pSIK1/SIK1 ratio, whose phosphorylation may inhibit ACC and FAS, was significantly reduced in the HFHF group (*p* < 0.001; Figure 5F). Lastly, the activities of glucose 6-phosphatase and malic enzyme, both sources of NADPH required for lipogenesis, remained unchanged under HFHF feeding (Figure 5G,H).

When evaluating the effect of *Opuntia stricta* var. *dillenii* peel extract, the low dose showed a significant reduction in FAS protein expression (Figure 5D), a significantly increased pSIK1/SIK1 ratio (*p* < 0.01; Figure 5F), and a slightly decreased malic enzyme activity (Figure 5H) relative to the HFHF group. In the H-OD group, only a significant reduction in FAS protein expression was observed (Figure 5D). Regarding *Opuntia ficus-indica* var. *colorada* pulp extract, the low dose significantly down-regulated ChREBP expression (Figure 5A) and demonstrated a tendency towards lower SREBP-1c levels (*p* = 0.06; Figure 5B) compared to the HFHF group. Additionally, this group demonstrated a significant rise in the pACC/ACC ratio (Figure 5C) and a significant reduction in FAS protein expression (Figure 5D). For the high dose, a significant elevation of the pACC/ACC ratio was also observed (Figure 5C), accompanied by a tendency towards reduced FAS protein expression (*p* = 0.07; Figure 5D) relative to the HFHF group. The remaining analysed parameters remained unchanged.

#### 3.3.3. Expression of Proteins Involved in Triglyceride Assembly

**Figure 6:**
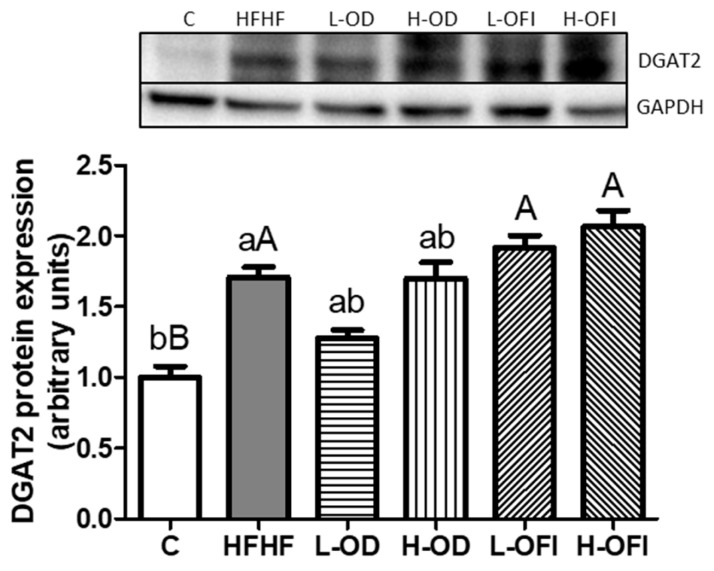
Hepatic protein expression levels of DGAT2 in rats fed a control diet (C), a high-fat high-fructose diet alone (HFHF), or supplemented with low or high doses of *Opuntia stricta* var. *dillenii* peel extract (L-OD and H-OD, respectively) or *Opuntia ficus-indica* var. *colorada* pulp extract (L-OFI and H-OFI, respectively) for 8 weeks. Data are presented as mean ± SEM. Differences among groups were analysed using one-way ANOVA followed by the Newman–Keuls *post hoc* test. Bars not sharing a common letter indicate statistically significant differences (*p* < 0.05). Lowercase letters denote comparisons among C, HFHF, L-OD, and H-OD groups, whereas uppercase letters denote comparisons among C, HFHF, L-OFI, and H-OFI groups. DGAT2: diacylglycerol O-acyltransferase 2.

Concerning triglyceride assembly, DGAT2 expression was significantly increased in the HFHF group compared to control animals (Figure 6). None of the *Opuntia* treatments altered this protein relative to the HFHF group.

#### 3.3.4. Enzymatic Activity and Protein Expression of Key Regulators Involved in Fatty Acid Oxidation and Mitochondrial Biogenesis

**Figure 7:**
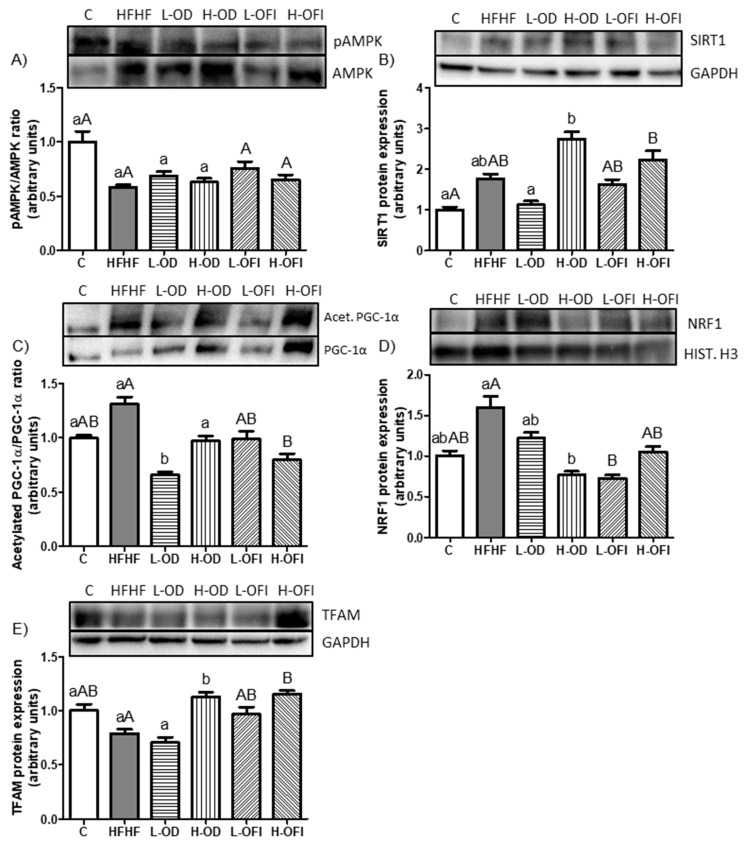
Hepatic protein expression levels of the pAMPK/AMPK ratio (**A**), SIRT1 (**B**), acetylated PGC-1α/PGC-1α ratio (**C**), NRF1 (**D**), and TFAM (**E**) in rats fed a control diet (C), a high-fat high-fructose diet alone (HFHF), or supplemented with low or high doses of *Opuntia stricta* var. *dillenii* peel extract (L-OD and H-OD, respectively) or *Opuntia ficus-indica* var. *colorada* pulp extract (L-OFI and H-OFI, respectively) for 8 weeks. Data are presented as mean ± SEM. Differences among groups were analysed using one-way ANOVA followed by the Newman–Keuls *post hoc* test. Bars not sharing a common letter indicate statistically significant differences (*p* < 0.05). Lowercase letters denote comparisons among C, HFHF, L-OD, and H-OD groups, whereas uppercase letters denote comparisons among C, HFHF, L-OFI, and H-OFI groups. AMPK: AMP-activated protein kinase; NRF1: nuclear respiratory factor 1; PGC-1α: peroxisome proliferator-activated receptor-gamma coactivator 1α; SIRT1: sirtuin-1; TFAM: mitochondrial transcription factor A.

Regarding energy metabolism, the pAMPK/AMPK ratio was reduced by 41.6% in the HFHF group compared to controls, although the difference was not statistically significant (Figure 7A). SIRT1 protein expression tended to be higher in HFHF-fed animals compared to the control group (*p* = 0.06; Figure 7B). For mitochondrial biogenesis, the acetylated PGC-1α/PGC-1α ratio and NRF1 and TFAM expression were analysed. The acetylated PGC-1α/PGC-1α ratio was higher in the HFHF group compared to the control group (*p* = 0.08; Figure 7C). NRF1 expression remained unchanged, although an increase of 60% was shown in the HFHF-fed animals relative to the control group (Figure 7D). TFAM protein expression was not significantly altered by the high-fat high-fructose diet (Figure 7E).

For *Opuntia stricta* var. *dillenii* peel extract, the L-OD group showed a significant decrease in the acetylated PGC-1α/PGC-1α ratio (*p* < 0.01; Figure 7C) compared to the HFHF treatment. By contrast, the administration of H-OD showed only a trend towards lower values (*p* = 0.08; Figure 7C). In addition, H-OD significantly decreased NRF1 protein expression (Figure 7D) and significantly increased TFAM levels (Figure 7E) compared to the HFHF group. Concerning *Opuntia ficus-indica* var. *colorada* pulp extract, the low dose showed a significant reduction in NRF1 expression (Figure 7D), while the high dose significantly decreased the acetylated PGC-1α/PGC-1α ratio (Figure 7C) and increased TFAM levels (Figure 7E).

**Figure 8:**
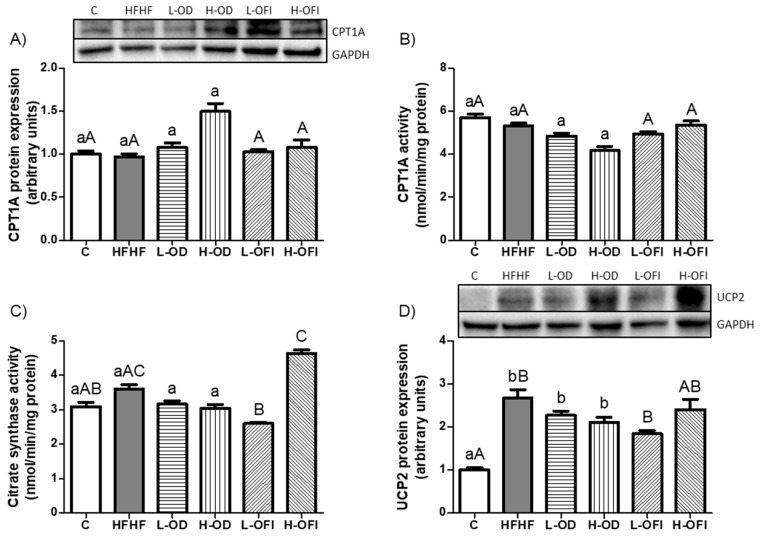
Hepatic protein expression levels of CPT1A (**A**), CPT1A activity (**B**), citrate synthase activity (**C**), and UCP2 protein expression (**D**) in rats fed a control diet (C), a high-fat high-fructose diet alone (HFHF), or supplemented with low or high doses of *Opuntia stricta* var. *dillenii* peel extract (L-OD and H-OD, respectively) or *Opuntia ficus-indica* var. *colorada* pulp extract (L-OFI and H-OFI, respectively). Data are presented as mean ± SEM. Differences among groups were analysed using one-way ANOVA followed by the Newman–Keuls *post hoc* test. Bars not sharing a common letter indicate statistically significant differences (*p* < 0.05). Lowercase letters denote comparisons among C, HFHF, L-OD, and H-OD groups, whereas uppercase letters denote comparisons among C, HFHF, L-OFI, and H-OFI groups. CPT1A: carnitine palmitoyl transferase-1a; UCP2: mitochondrial uncoupling protein 2.

Hepatic fatty acid oxidation was evaluated by measuring CPT1A protein expression and activity, both of which remained unchanged with the HFHF feeding compared to the control group (Figure 8A,B). Citrate synthase activity, used as an indicator of mitochondrial density, also showed non-statistical differences between the HFHF group and the control group (Figure 8C). In contrast, UCP2 expression was significantly increased in the HFHF group compared to the control group (Figure 8D).

Regarding *Opuntia stricta* var. *dillenii* peel extract, only a tendency towards higher values in CPT1A expression was reported in the H-OD group compared to the HFHF cohort (*p* = 0.06; Figure 8A). For *Opuntia ficus-indica* var. *colorada* pulp extract, the low dose significantly decreased citrate synthase activity, while the high dose exhibited a trend towards higher levels in this protein expression relative to the HFHF group (*p* = 0.08; Figure 8C).

#### 3.3.5. Expression of Proteins Involved in Lipolysis and Hepatic Lipid Release

**Figure 9:**
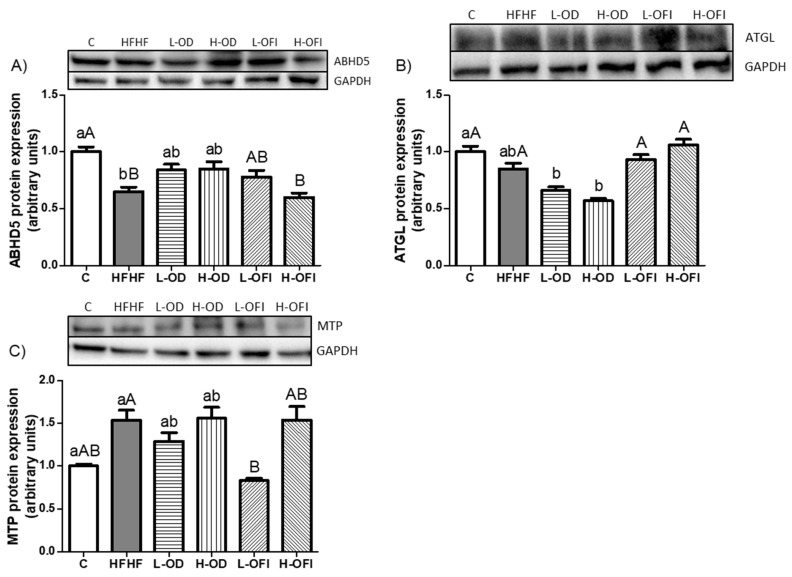
Hepatic protein expression levels of ABHD5 (**A**), ATGL (**B**), and MTP (**C**) in rats fed a control diet (C), a high-fat high-fructose diet alone (HFHF), or supplemented with low or high doses of *Opuntia stricta* var. *dillenii* peel extract (L-OD and H-OD, respectively) or *Opuntia ficus-indica* var. *colorada* pulp extract (L-OFI and H-OFI, respectively) for 8 weeks. Data are presented as mean ± SEM. Differences among groups were analysed using one-way ANOVA followed by the Newman–Keuls *post hoc* test. Bars not sharing a common letter indicate statistically significant differences (*p* < 0.05). Lowercase letters denote comparisons among C, HFHF, L-OD, and H-OD groups, whereas uppercase letters denote comparisons among C, HFHF, L-OFI, and H-OFI groups. ABHD5: abhydrolase domain containing 5, lysophosphatidic acid acyltransferase; ATGL: adipose triglyceride lipase; MTP: microsomal triglyceride transfer protein.

To evaluate the lipolysis pathway, hepatic ABHD5 protein expression (also named comparative gene identification-58, CGI-58), a coactivator of ATGL lipase, was measured [31]. ABHD5 expression was significantly diminished in the HFHF group compared to the control group (Figure 9A), whereas ATGL protein expression remained unchanged (Figure 9B). Hepatic lipid release was assessed by measuring MTP protein expression, which showed a tendency towards higher values in the HFHF group compared to the control group, with an increase of 53.5% (*p* = 0.08; Figure 9C).

For *Opuntia stricta* var. *dillenii* peel extract, the H-OD group showed a tendency towards reduced ATGL expression compared to the HFHF group (*p* = 0.06; Figure 9B). Concerning *Opuntia ficus-indica* var. *colorada* pulp extract, the low dose significantly reduced MTP protein expression relative to the HFHF treatment (Figure 9C).

### 3.4. Expression of Proteins Related to Autophagy

**Figure 10:**
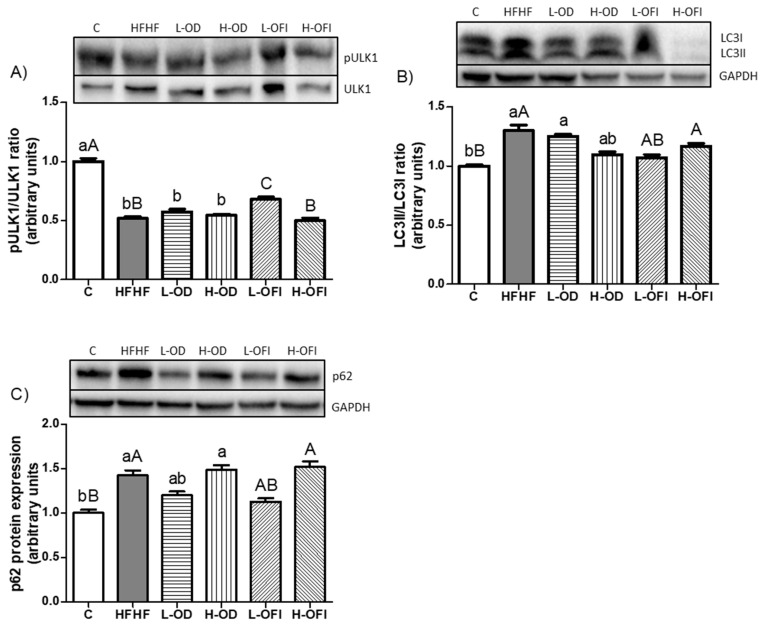
Hepatic protein expression levels of pULK1/ULK1 ratio (**A**), LC3II/LC3I ratio (**B**), and p62 (**C**) in rats fed a control diet (C), a high-fat high-fructose diet alone (HFHF), or supplemented with low or high doses of *Opuntia stricta* var. *dillenii* peel extract (L-OD and H-OD, respectively) or *Opuntia ficus-indica* var. *colorada* pulp extract (L-OFI and H-OFI, respectively) for 8 weeks. Data are presented as mean ± SEM. Differences among groups were analysed using one-way ANOVA followed by the Newman–Keuls *post hoc* test. Bars not sharing a common letter indicate statistically significant differences (*p* < 0.05). Lowercase letters denote comparisons among C, HFHF, L-OD, and H-OD groups, whereas uppercase letters denote comparisons among C, HFHF, L-OFI, and H-OFI groups. LC3: microtubule-associated protein 1A/1B-light chain 3; p62: sequestosome-1; ULK1: unc-51-like autophagy activating kinase 1.

Autophagy was examined by analysing the expression of key proteins associated with this pathway. Phosphorylation of ULK1 at serine 757, which inhibits this protein and consequently autophagy, was assessed, showing a significant reduction in the pULK1/ULK1 ratio in the HFHF group (Figure 10A). Additionally, the LC3II/LC3I ratio and p62 protein expression, markers of autophagosome formation and autophagy flux, respectively, were significantly augmented in HFHF-fed animals (Figure 10B,C).

In the case of *Opuntia stricta* var. *dillenii* peel extract, none of the tested doses influenced the parameters involved in autophagy. By contrast, the low dose of *Opuntia ficus-indica* var. *colorada* pulp extract significantly increased the pULK1/ULK1 ratio relative to the HFHF group (Figure 10A). Additionally, the L-OFI group demonstrated a tendency towards reduced LC3II/LC3I ratio (*p* = 0.1; Figure 10B) and p62 protein expression (*p* = 0.06; Figure 10C).

## 4. Discussion

The Western diet, rich in saturated fatty acids and sugars, has been linked to elevated risk of MASLD [32]. More recently, chronic fructose intake has been implicated as a contributor to MASLD [33]. On this basis, several studies have employed the high-fat high-fructose diet to establish a steatosis model in rats [34,35,36]. In the present study, the administration of a high-fat high-fructose diet for eight weeks led to an increase in liver weight together with liver steatosis development. This was confirmed by the elevation of hepatic triglyceride content and histological analysis. These data indicate the successful establishment of a diet-induced MASLD model.

Among the four treatments analysed in the present study, only the pulp extract of *Opuntia ficus-indica* var. *colorada* at the low dose (25 mg/kg body weight/day) partially prevented liver steatosis. At the higher dose (100 mg/kg body weight/day), a reduction in triglyceride values was suggested. This liver lipid-lowering of *Opuntia* extracts has been described in other studies employing material from various plan parts. Examples include the study by Morán-Ramos et al. [37], who examined the effect of a cladode extract in Zucker rats on a standard diet, and the work of Kang et al. [38], who studied the effect of a DWJ504 seed extract in C57BL7/6 mice on a high-fat diet. Similar to the present study, other reports indicate that higher doses of *Opuntia* extracts do not necessarily produce stronger effects. For example, Kang et al. evaluated various concentrations of the DWJ504 seed extract and found that the highest dose did not result in the greatest reduction in hepatic triglyceride levels. The anti-steatotic effect of *Opuntia ficus-indica* extract in the present study was also supported by histological analysis. In line with these results, supplementation with *Opuntia ficus-indica* fruit vinegars or seed extracts in rodents fed a high-fat diet has been demonstrated to attenuate histopathological damage [38,39].

With respect to the lack of effect of *Opuntia stricta* var. *dillenii* peel extract, comparison with the existing literature is not possible, as no previous data have been reported. In accordance with this result, histopathological analysis showed that administration of this *Opuntia* extract did not substantially modify the grade of steatosis relative to the HFHF group. In contrast, studies inducing liver damage with chemicals have described improvements following supplementation with *Opuntia stricta* var. *dillenii* cladode juice or fruit hydroalcoholic extract [40,41]. It is noteworthy that in the study employing the fruit hydroalcoholic extract, the lower dose (100 mg of extract/kg body weight), which corresponds to one of the doses tested in the present study, did not alleviate histological damage, and hepatoprotective effects were only observed at the higher dose (200 mg of extract/kg body weight) [41]. These observations suggest that higher doses of *Opuntia stricta* var. *dillenii* peel extract may be required compared to *Opuntia ficus-indica* pulp extract to achieve hepatoprotective effects.

Therefore, after demonstrating that *Opuntia ficus-indica* var. *colorada* pulp extract reduced liver lipid accumulation and improved histological indicators, and in line with the aim of the present study, the next step was to analyse the potential mechanisms of action underlying these effects. To this end, changes in metabolic pathways related to hepatic lipid “input” and hepatic lipid “output” were evaluated. Although an anti-steatotic effect was only detected with the low dose of the extract, metabolic pathways were analysed in all experimental groups to identify early alterations that, under longer treatment durations, might contribute to reductions in hepatic triglyceride content.

Regarding hepatic lipid uptake, protein expression of FATP2, FATP5, and CD36 was measured, since circulating fatty acid entry into the liver is primarily mediated by these transporters [42]. Administration of the high-fat high-fructose diet caused an increase in CD36 protein expression, indicating enhanced fatty acid influx into the liver. Concerning *de novo* lipogenesis, the reduction in the pACC/ACC protein ratio shows that ACC activity was increased, because this enzyme is inhibited by phosphorylation. In the case of FAS, no changes in its activity were observed. The apparent discrepancy between this result and the increase in FAS protein expression can be justified by the fact that the enzyme activity does not depend solely on the amount of protein. Indeed, multiple factors, such as post-translational modifications, regulate enzyme function. This situation has been also reported by other authors [43]. On the other hand, phosphorylated SIK1 can inhibit lipogenesis [44], and in the present study, the pSIK1/SIK1 ratio was decreased in the HFHF group. Taking into account that ACC is the rate-limiting enzyme in *de novo* lipogenesis, altogether these results can allow us to propose a potential increase in this metabolic pathway.

A result that can be surprising is the reduction observed in ChERBP protein expression, because in a great number of the reported studies a significant increase is observed. This fact can be attributable to the specific isoform detected. High-carbohydrate diets induce a marked increase in hepatic ChREBPβ expression, but not in ChREBPα levels. Whereas in the present study the isoform ChREBPα was measured, in the vast majority of the published studies, the specific ChREBP isoform detected by Western blot analysis was not clearly specified. ChREBPα isoform is generally considered a more robust indicator of ChREBP protein expression, as it functions as a nutrient sensor. In this line, other authors have reported an increase in *de novo* lipogenesis without changes in ChREBPα [45,46]. Regarding DGAT2, its protein expression was increased in this group, suggesting greater triglyceride assembly [47].

With regard to triglyceride output, processes such as mitochondrial β-oxidation, triglyceride mobilisation, and triglyceride packaging with apolipoprotein B (ApoB) for secretion into the bloodstream as very low-density lipoproteins (VLDLs) [48] should be considered. As for fatty acid oxidation, CPT1A serves as a rate-limiting enzyme in β-oxidation by controlling the mitochondrial import of fatty acids [49]. Several proteins influence this pathway. AMPK directly phosphorylates ACC, thereby inhibiting *de novo* lipogenesis and reducing malonyl-CoA levels, which act as allosteric inhibitors of CPT1A [50]. AMPK also contributes to PGC-1α activation through phosphorylation [51]. Therefore, it can be inferred that AMPK activation leads to an enhancement of β-oxidation. Moreover, SIRT1 can deacetylate PGC-1α, promoting hepatic fatty acid β-oxidation [48]. In the present study, no significant changes were observed in parameters related to this metabolic pathway in the HFHF group.

Regarding lipolysis, ATGL is a key lipase involved in hepatic triglyceride mobilisation [52]. ABHD5 can regulate lipolysis by activating ATGL [53]. In the HFHF group, ABHD5 protein expression was reduced, while ATGL protein levels remained unchanged, suggesting a potential decrease in the activation of this lipase. Triglyceride secretion is regulated by MTP, which integrates triglycerides into ApoB and serves as a crucial enzyme for the assembly and release of VLDL from hepatocytes [54]. In the HFHF group, MTP protein expression tended to be higher compared to the control group, which may be related to the elevated content of saturated fatty acids in the diet [54].

In summary, high-fat high-fructose diet was associated with the advancement of hepatic steatosis, involving fatty acid uptake and triglyceride assembly, collectively contributing to enhanced triglyceride formation. The contribution of increased *de novo* lipogenesis cannot be ruled out. Decreased lipolysis may also have played a role.

In addition to the assessment of hepatic triglyceride metabolism, several proteins involved in autophagy were measured. Autophagy in the liver is widely recognised as a mechanism for maintaining cellular and metabolic homeostasis [55]. Recent studies suggest that autophagy plays a crucial role in the removal of lipid droplets from hepatocytes, indicating its involvement in the regulation of MASLD development [56]. Autophagy has also been related to an increase in oxidative stress, DNA damage, and endoplasmic reticulum stress, which can ultimately lead to cell death [57]. In short, autophagy initiation begins with phosphorylation of the ULK1 complex, followed by LC3I activation during phagophore expansion to form LC3II. Lastly, p62 serves as a receptor that connects ubiquitinated proteins to LC3 and delivers them to autophagosomes [58]. In the present study, the HFHF group exhibited increased LC3II/LC3I ratio and elevated p62 protein expression, indicating increased autophagy. Obviously, this effect is not a mechanism of action justifying the induced steatosis, because the expected change would have been a decrease in protein expression. On the contrary, under feeding conditions that led to negative metabolic situations (steatosis and oxidative stress [12]), such as steatosis in our study, adaptive responses take place in order to limit the problem.

The *Opuntia ficus-indica* extract that prevented hepatic steatosis induced a significant reduction in FATP2, a fatty acid transporter, at both doses. This outcome is in line with a previous study reported by our group, where an *in vitro* model of murine hepatic organoid revealed down-regulation of fatty acid transporter genes [16]. Both doses of the extract prevented the decrease in the pACC/ACC ratio induced by the HFHF diet, and thus a potential contribution of decreased *de novo* lipogenesis cannot be ruled out. A lower availability of fatty acids for triglyceride synthesis can be proposed as one mechanism of action involved in the preventive effects of the extract on steatosis. Although studies reported in the literature have shown reductions in *de novo* lipogenesis induced by *Opuntia ficus-indica* extracts, these results are not comparable with those obtained in the present work because different extracts have been used. Thus, whereas in the reported studies extracts obtained from cladodes or seeds were administered to animals, in the present study fruit extracts were provided [38,59]. Very important differences in terms of bioactive compounds are found between *Opuntia* cladodes, seeds, and fruits. In the present study, no change in DGAT2 expression was detected, indicating that triglyceride assembly was unaffected by this extract.

With respect to mitochondriogenesis and fatty acid oxidation, no clear effects of the extract were detected on this metabolic pathway, similar to the results obtained for lipolysis. Few studies have analysed the influence of *Opuntia* on hepatic fatty acid oxidation. Two studies reported an increase in β-oxidation through the up-regulation of CPT1A: one using *Opuntia ficus-indica* cladodes in Zucker obese rats for seven weeks [37] and another using cladodes in male Wistar rats fed a high-fat high-sucrose diet for one month [59]. The discrepancy with the present study may relate to differences in the *Opuntia* preparation (fruit pulp versus cladodes) or experimental design. Consistent with our results, supplementation of high-fat-diet-fed mice with an *Opuntia ficus-indica* seed extract for ten weeks did not alter CPT1A protein expression [38].

Lastly, MTP activity was measured to evaluate triglyceride output from the liver, and a significant decrease was shown only in rats receiving the low dose of the extract. This effect is unlikely to represent a direct mechanism for the anti-steatotic action of the extract but may reflect the lower liver triglyceride formation in the L-OFI group. A similar relationship has been described with other compounds. For instance, treatment with S-nitroso-N-acetylcysteine, a natural compound with anti-steatotic properties, led to histological improvement and reduced MTP protein expression in mice, an effect attributed to decreased fatty acid biosynthesis [60]. Moreover, the reduction in MTP content resulting from decreased fatty acid synthesis has been associated with ChREBP down-regulation [61], which was also detected in the L-OFI experimental group.

With respect to autophagy, in general terms no clear changes were observed in rats treated with *Opuntia ficus-indica* extracts.

**Figure 11:**
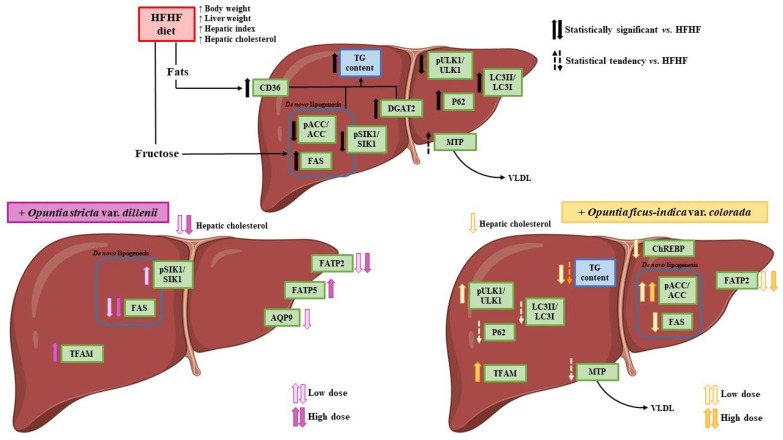
Schematic illustration of the mechanisms by which *Opuntia ficus-indica* var. *colorada* and *Opuntia stricta* var. *dillenii* act to prevent diet-induced hepatic steatosis.

It is noteworthy that both doses of the *Opuntia ficus-indica* extract affected the same metabolic processes, although a significant decrease in liver steatosis was detected only with the low dose (Figure 11). This outcome can be explained by the comparable magnitude of steatosis reduction between the two treatments (−12.5% vs. −8.9%), despite the statistical difference.

For the *Opuntia stricta* var. *dillenii* extract, the only pathway clearly affected was fatty acid uptake, reflected by a reduction in FATP2 protein expression. This isolated effect was likely insufficient to reduce hepatic steatosis, as indicated by both the histological analysis and liver triglyceride content.

The main limitation of the present work is that the involvement of some potential mechanisms cannot be totally accepted or ruled out. In order to do that, additional physiological determinations, such as the direct measurement of *de novo* lipogenesis, or a lipidomic analysis, should be addressed. Moreover, this work reports preclinical effects, which need to be confirmed in further studies involving human beings.

## 5. Conclusions

In summary, under the conditions of the present study, the pulp extract of *Opuntia ficus-indica* var. *colorada*, but not the peel extract of *Opuntia stricta* var. *dillenii*, effectively prevents hepatic steatosis. Although the reduction in triglyceride accumulation of *Opuntia ficus-indica* extract is modest, this effect, together with its previously demonstrated antioxidant and anti-inflammatory properties, highlights its potential as a preventive strategy for MASLD. The anti-steatotic effect of *Opuntia ficus-indica* extract mainly results from decreased liver fatty acid uptake, leading to reduced fatty acid availability for triglyceride synthesis.

## Figures and Tables

**Table 1:** Major phenolic compounds and betalains in the peel extract of *Opuntia stricta* var. *dillenii* and the pulp extract of *Opuntia ficus-indica* var. *colorada*.

Compound	Content (µg of Compound/g Dry Weight)	
	*Opuntia stricta* var. *dillenii* Peel Extract	*Opuntia ficus-indica* var. *colorada* Pulp Extract
Portulacaxanthin III (Bx-glycine)	n.d.	30 ± 1.6
Vulgaxanthin III (Bx-asparagine)	n.d.	14.6 ± 0.9
Vulgaxanthin I (Bx-glutamine)	n.d.	12.4 ± 0.4
Vulgaxanthin II (Bx-glutamic acid)	n.d.	18 ± 2.9
Indicaxanthin (Bx-proline)	n.d.	510 ± 14
Betanin	5160 ± 88	tr.
Isobetanin	3038 ± 95	n.d.
2′-*O*-apiosyl-4-*O*-phyllocactin	1372 ± 102	n.d.
**5″-*O*-E-sinapoyl-2-apiosyl-phyllocactin**	183 ± 37	n.d.
Neobetanin	429 ± 11	n.d.
Piscidic acid	19,269 ± 382	2564 ± 108
**Quercetin-3-*O*-rhamnosyl-rutinoside (QG3)**	202 ± 6	n.d.
Quercetin glycoside 2 (QG2)	245 ± 9	14.4 ± 3.3
Isorhamnetin glucoxyl-rhamnosyl-pentoside (IG2)	989 ± 21	30.4 ± 3.9

n.d.: not detected; tr.: traces.

**Table 2:** Nutritional composition of experimental diets.

	STD	HFHF
Total energy (kcal/g)	3.9	4.5
Carbohydrates (energy %)	63.9	40
Fructose (energy %)	-	10
Proteins (energy %)	20.3	20
Lipids (energy %)	15.8	40

HFHF: high-fat high-fructose diet; kcal: kilocalories; STD: standard diet.

## Data Availability

The original contributions presented in this study are included in the article. Further inquiries can be directed to the corresponding author.

## Abbreviations

The following abbreviations are used in this manuscript:

ABHD5	Abhydrolase domain containing 5, lysophosphatidic acid acyltransferase
ACC	Acetyl-CoA carboxylase
AMPK	AMP-activated protein kinase
ApoB	Apolipoprotein B
AQP9	Aquaporin 9
ATGL	Adipose triglyceride lipase
BSA	Bovine serum albumin
CD36	Cluster of differentiation 36
ChREBP	Carbohydrate-responsive element-binding protein
CPT1A	Carnitine palmitoyl transferase-1a
DGAT2	Diacylglycerol O-acyltransferase
FAS	Fatty acid synthase
FATP2	Fatty acid transport protein 2
FATP5	Fatty acid transport protein 5
GAPDH	Glyceraldehyde 3-phosphte dehydrogenase
HCC	Hepatocellular carcinoma
HFHF	High-fat high-fructose diet
LC3	Microtubule-associated protein 1A/1B-light chain 3
MASLD	Metabolic dysfunction-associated fatty liver disease
MASH	Metabolic dysfunction-associated steatohepatitis
MTP	Microsomal triglyceride transfer protein
NADPH	Nicotinamide adenine dinucleotide phosphate
NRF1	Nuclear respiratory factor 1
PGC-1α	Peroxisome proliferator-activated receptor-gamma coactivator-1α
p62	Sequestosome-1
PVDF	Polyvinylidene difluoride
SIK1	Salt inducible kinase 1
SIRT1	Sirtuin-1
SREBP-1c	Sterol regulatory element-binding transcription factor 1
TFAM	Mitochondrial transcription factor A
TG	Triglyceride
UCP2	Mitochondrial uncoupling protein 2
ULK1	Unc-51-like autophagy activating kinase 1
VLDLs	Very low-density lipoproteins
